# Global Inequality in Service Sector Development: Trend and Convergence

**DOI:** 10.3389/fpsyg.2021.792950

**Published:** 2021-11-25

**Authors:** Ning Ma, Wai Yan Shum, Tingting Han, Tsun Se Cheong

**Affiliations:** ^1^School of Financial Management, Hainan College of Economics and Business, Haikou, China; ^2^Department of Economics and Finance, The Hang Seng University of Hong Kong, Shatin, Hong Kong SAR, China

**Keywords:** COVID-19 pandemic, service sector development, distribution dynamics analysis, disparity, convergence

## Abstract

The COVID-19 pandemic has caused a huge impact on global service sector. In the pandemic background, to understand the disparity in service sector outputs at the global level is crucial for assessing the effectiveness of development policies in different countries. This study investigate the global service sector and it aims to investigate the transitional dynamics of the output from the service sector by adopting stochastic kernel analyses. Distribution dynamics analyses are carried out for all the countries in the world. The data are then divided into different regional and income groups to evaluate the impacts of geographical location and income on the development of the service sector. The results show that the Global North will continue to make greater strides, while the output capacity in many Global South countries struggles to reach the global average. Moreover, it is shown that countries with higher per capita income will perform better in the development of their service sector than those with low per capita income, thereby highlighting the persistence of global inequality. Finally, this study shows that the Sub-Saharan Africa region and the South Asia region both are very important in the alleviation of global inequality.

## Introduction

Coronavirus disease 2019 (COVID-19) continues to appear globally. As of October 08, 2021, the number of infected people globally is almost 237 million and it has caused more than 4.8 million deaths worldwide (National Health Commission [NHC], 2021). As stated by Goodell (2020), the pandemic may cause economic havoc through its heavy economic costs. COVID-19 related closures and lockdowns have a larger knock-on effect on global service sector and it is worth to investigate the disparity in service sector outputs.

**FIGURE 1:**
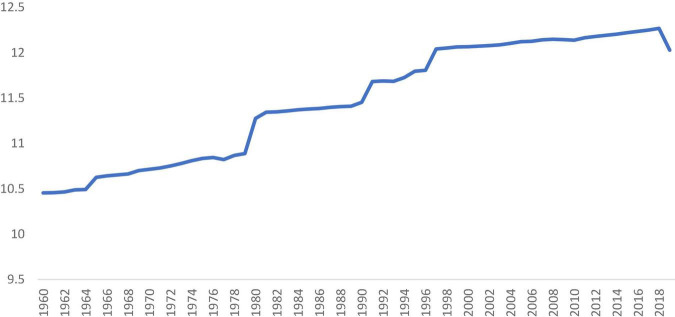
Log service value added. Data reflect available observations from all countries for 1960–2019 and are reported in constant 2010 US $. Source: World Bank (2021).

**FIGURE 2:**
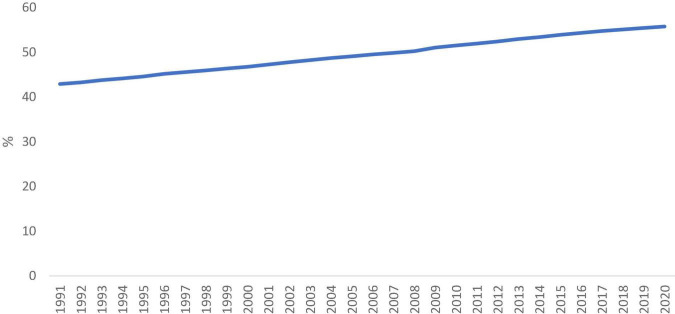
Employment in service (% of total employment). Source: World Bank (2021).

Structural transformation is an integral part of the economic growth and development process. During the industrialization process, the share of agriculture in employment and output declines, and the share of manufacturing and service increases. While the manufacturing sector matures, the employment and output in the service sector continue to grow. In some highly open countries, comparative advantage is strongly concentrated in manufacturing, however, the output of manufacturing may decline due to the economy eventually rebalances in the increase in service component (Noland et al., 2012). According to Figures 1, 2, service sector output and employment share in the world average level were increased significantly during the past decades.

However, the disparity in the service sector growth in the world also increased dramatically in that period. Ogundele et al. (2020) confirmed inequality in the health care service sector in Sub-Saharan Africa. Antonelli and Tubiana (2020) found that knowledge-intensive structural change raised the levels of income inequality in 20 OECD countries from 1990 to 2016. Mehta and Hasan (2012) examined the effects of trade and service liberalization on wage inequality in India. They found that 30–66% of the increased wage inequality was due to changes in industry wage. Mookerjee and Kalipioni (2010) used a sample of developing and developed countries to study the finance service sector and income inequality. The authors concluded that barriers to bank access significantly increase income inequality. Xiao et al. (2019) examined the CO_2_ emission performance of 25 Yangtze River Delta cities during 2007–2016. They found that the total factor carbon emission performance (TCPI) gap between the secondary and service industries narrowed over the study period and TCPI inequality in secondary industry was much larger than in the service industry. Some other scholars have focused on examining the convergence of the service sector. For example, Kinfemichael and Morshed (2018) examined the unconditional convergence of labor productivity in the service sector for 95 countries. Wang and Zhang (2014) examined the σ-convergence, stochastic convergence, and β-convergence of CO_2_ emission in different service sub-sector in China. The authors found that per capita emission in all sectors converged across provinces from 1996 to 2010. However, most of the studies on inequality in the service sector have been based on provincial-level or single-country data, and thus the policy implications derived from these studies are valid for formulating policies at the country level and the provincial level only.

The existing literature cannot provide policy makers with relevant information on service sector inequality. This study aims to fill the gap in the literature by studying the disparity in the service sector growth on a global scale. The database used in this study is made up of countries in the world and this is the most comprehensive research ever undertaken at the global level for investigating disparity and convergence of service sector output by using distribution analysis. This is the first study to uses a new stochastic kernel approach in the transition dynamics analysis, which provides an in-depth understanding of the disparity and convergence feasibility of service sector output around the globe. Moreover, this study proposes a new analytical framework for interpreting mobility probability, which can be used in conjunction with a contour map or three-dimensional plot. The proposed new framework offers additional insights and greatly enhances the traditional distribution dynamics analysis (Quah, 1993a,b, 1996a,b,c, 1997).

In this study, the causal relationship between the impact of COVID-19 and global service sector inequality is not our priority to study and we are focused on the distribution of global service inequality. COVID-19 has spread all over the world and the social distancing rule has forced many service firms closed. This has significantly affected the global service sector and exacerbated the inequality in the service sector. A comprehensive study on the distribution of the Relative Service Value-Added per Capita (RSVAPC) of global countries provides policy makers with reliable references to improve global service sector development strategies by prioritizing supportive policies across the countries. The world organization for service development can encourage capital and technology resources directed to these lagging countries with imbalance RSVAPC. Moreover, the distribution dynamics approach would forecast the future pattern of country-level RSVAPC, this will show the United Nations and the governments an effective way to promote the rationalization of RSVAPC transition.

The rest of the paper begins with a brief review of the literature in Section “Literature Review.” Section “Data and Methodology” describes the data and methodology. Section “Discussions” investigates the dynamics of the spatial distribution of RSVAPC, while Section “Conclusion and Implications” summarizes the findings and discusses the policy implications.

## Literature Review

In fact, most of the literature on the rising size of the service sector has emphasized its growth, rather than distributional, effects (Cavelaars, 2006; Chakraborty and Nunnenkamp, 2008; Singh, 2014; Hassan and Abdullah, 2015; de Souza et al., 2016; Freytag and Fricke, 2017; Latorre et al., 2018; Omri, 2018; Basuil and Datta, 2019; Beqiraj et al., 2019; Doytch and Uctum, 2019; Morikawa, 2019; Liao, 2020). These studies all confirms that degree of competition, FDI, innovation and oil revenue is positively correlated with the growth of service sector.

However, other scholars have focused on the study of productivity in service sector. Kuijs and Wang (2006) suggests that contribution to increased service sector productivity comes from growth in capita per worker and TFP growth. Their argument is widely accepted, and many researchers suggest that fast productivity growth in the service sector contribute to the balance growth of economies (Wu, 2007; Bosworth and Collins, 2008; Miroudot et al., 2012; Nabar and Yan, 2013; Sauian et al., 2013; Tochkov and Yu, 2013; García-Pozo et al., 2018; Karim et al., 2018; Kinfemichael and Morshed, 2018; Lee and McKibbin, 2018; Zhang and Lin, 2018; Álvarez-Rodríguez et al., 2019; Haller and Lyons, 2019).

In recent decade, the relationship between CO2 emissions, energy, and the service sector has become a hot topic. Input-output approach and the data envelopment analysis (DEA) approach have been applied frequently for study the CO2 emissions and energy efficiency on service sector (Gowdy and Miller, 1987; Alcántara and Padilla, 2009; Butnar and Llop, 2011; Fang et al., 2013; Lin and Zhang, 2017; Brown et al., 2020; Hou et al., 2020; Wang et al., 2020). Some researchers have examined the energy consumption on service sector. For example, Mulder et al. (2014) analyzed the energy intensity developments across 23 service sectors in 18 OECD countries over the period 1980–2005. Tang and Shahbaz (2013) used the annual data from 1972 to 2010 to assess the causal relationship between electricity consumption and real output at the aggregate and sectoral levels in Pakistan. Voulis et al. (2017) used Netherlands as the case to study the impact of the service sector on the potential for renewable resource integration in urban energy systems. Xing et al. (2018) estimated the energy demand in the Chinese service sector at the provincial level up to 2030.

In terms of methodology, due to the problems of multicollinearity, a major shortcoming is that regression models cannot include many independent variables. This would cause many other relevant factors are neglected. Also, the output of regression models is the forecasted value of the dependent variable. This cannot be used to forecast the evolution of distribution. Moreover, regression models fail to provide information on the value of the convergence and the number of convergence clubs in the distribution (Shi et al., 2020). However, this information is important for policy makers. The distribution dynamics is making no assumptions about the underlying distribution of the population and it allows us to understand the transitional dynamics of RSVAPC over time. This method could provide intra-distribution mobility for the countries. It can even offer detailed information on each spatial grouping by revealing their distinguishing features in terms of RSVAPC (Cheong et al., 2019). In addition, this approach can be used to offer a forecast for the shape of the distribution of RSVAPC in the long run, which is an issue particularly relevant from the policy implications perspective.

A noteworthy feature of this strain of research is that most of the existing studies are based on single country data or only focused on few countries and there has been no application of a global scale. In addition, the distribution dynamics approach has not been employed in service sector studies. Compare with existing convergence methods, the distribution dynamic approach does not need assumptions about the underlying distribution of the population, and it also has some advantages over the parametric methods. Thus, this paper aims to study the evolution and convergence of RSVAPC on a global scale.

## Data and Methodology

The dataset was compiled from the World Development Indicators provided by the World Bank. The data of value-added (constant 2010 US$) of the service sector and the population of each country were gathered first and the value-added per capita was computed accordingly. After that, the global average value of value-added per capita was derived for every year. The value of each country’s value-added per capita was then divided by the global average to derive the relative service value-added per capita (RSVAPC) for this particular year for every country one by one. Almost all the countries have been included in this study although a few of them are missing due to data unavailability.

Distribution dynamics analyses were then conducted based on the RSVAPC values of each country. It is worth noting that the distribution dynamics approach was first proposed by Quah (1993a) in the 1990s. This approach focuses on the evolution of the distribution across time so that it is very useful for investigating the changes in distribution and the underlying trend behind these changes.

The distribution dynamics approach is different from the econometrics analysis which is commonly employed in research. First, econometric analysis can only offer the forecast of the value of the dependent variable which is a forecast of a point, so it is impossible to prepare a forecast of the shape of a distribution. On the contrary, distribution dynamics analysis can be used to evaluate the change in the distribution in the long run. Moreover, the probability of the movement of the entities within a distribution can also be computed by the distribution dynamics approach so pragmatic policy suggestions can be derived and the underlying trend across time can be known in detail.

The distribution dynamics analysis approach can be divided into two categories, namely, the Markov transition matrix analysis and the stochastic kernel analysis. It is noteworthy that the latter is deemed to be much better than the former because the latter is not affected by the problem of arbitrariness in grid selection, and the values of demarcation of state for the latter can be derived objectively. As a result, the stochastic kernel approach is employed in this study.

The bivariate kernel estimator can be represented by the format as shown below:


(1) $$\hat{f}\,\left(x,y\right)=\frac{1}{n\,h_{1}\,h_{2}}\,\sum_{i=1}^{n}K\,\left(\frac{x-X_{i,t}}{h_{1}},\frac{y-X_{i,t+1}}{h_{2}}\right)$$

where *h*_1_ and *h*_2_ are values of bandwidth. They are optimal bandwidths which were computed by the procedure suggested by Silverman (1986), *X*_*i,t*_ is an observed value of RSVAPC at time *t*, *x* is RSVAPC at time *t*, *X_*i,t*+1_* is the observed value of RSVAPC at time *t*+1, *y* is RSVAPC at time *t*+1, *K* is the normal density function, and *n* is the total number of observations.

Given that the data of RSVAPC are not evenly distributed throughout the dataset, therefore, the number of data varies greatly across the range of RSVAPC. Many observations may congregate around some specific values of RSVAPC, whilst other values may only have a few data. This can lead to spareness in the dataset which can affect the estimation, therefore, a two-step procedure suggested by Silverman (1986) was employed to take this into consideration. In the first stage, a pilot estimate was made and the bandwidth was rescaled in the second stage based on the spareness of the data.

Assuming that the evolution of data is time-invariant and first order, the distribution dynamics can be represented by:


(2) $$f_{t+\tau }\left(z\right)=\int_{0}^{\infty }g_{\tau }\left(z|x\right)f_{t}\left(x\right)dx$$

where *g*_τ_(*z*|*x*) is the probability kernel which maps the distribution from time *t* to *t+τ*, *f*_*t* + τ_(*z*) is the τ-period-ahead density function of *z* conditional on *x*. and *f*_*t*_(*x*) is the kernel density function of the distribution of RSAPC at time *t*.

By repeated use of Eq. 2, the ergodic distribution (given that it exists) can be derived by:


(3) $$f_{\infty }\left(z\right)=\int_{0}^{\infty }g_{\tau }\left(z|x\right)f_{\infty }\left(x\right)dx$$

where *f*(*z*) is the ergodic density function which is the steady-state distribution in the long run.

Cheong and Wu (2018) developed the Mobility Probability Plot (MPP) for studying the probability of future movement of the entities within a distribution. This is a very useful tool and it has been applied extensively in many different fields, for example, industrial output (Cheong and Wu, 2018), carbon emissions (Cheong et al., 2016; Zhang et al., 2020), and consumption of electricity (Cheong et al., 2019).

The MPP is defined as *p*(*x*) which is the net probability of moving upward for the countries within the distribution:


(4) $$p\left(x\right)=\int_{x}^{\infty }g_{\tau }\left(z|x\right)dz-\int_{0}^{x}g_{\tau }\left(z|x\right)dz$$

The MPP indicates the net probability of an increase in the RSVAPC for different countries. It is denoted in percentage and the value is from -100 to 100. A negative value implies that the country has a higher tendency to move downward within the distribution in the next period, while a positive value of MPP indicates that the country has a higher tendency to move upward within the distribution.

## Discussion

For a long, the evolution of a country or the whole world, from agricultural to industrial and then to a service economy, seems to be a natural and inevitable process for economic development. The service sector remains a massive component of the economy in developed countries such as the United States and the United Kingdom, accounting for about 80% of GDP, as per the World Bank National Accounts Data. On the contrary, the figure declines to nearly 30% in low-income countries. Despite the difference, it is noticeable the share of this sector in both developed and developing economies is increasing with each passing year. Moreover, jobs in the service industry are expected to boost employment growth in the future; while the rate of employment in agriculture and manufacturing industries will be on a decline, according to the Kühn et al. (2018) World Employment and Social Outlook.

This section will present global development in the service sector analyzed through the distribution dynamic model. The analyzed data can show an all-inclusive pattern of development in the service sectors of all countries across the globe. This will further reveal the disparity in the service sector growth and the viability to achieve convergence gradually. For further analysis of this critical issue in detail, the full dataset was divided into different groupings. Moreover, to ensure an in-depth analysis, all distribution dynamics analyses were performed separately for each grouping. The groupings are determined based on the Global North and Global South, levels of income, and geographical locations.

### All Countries

**FIGURE 3:**
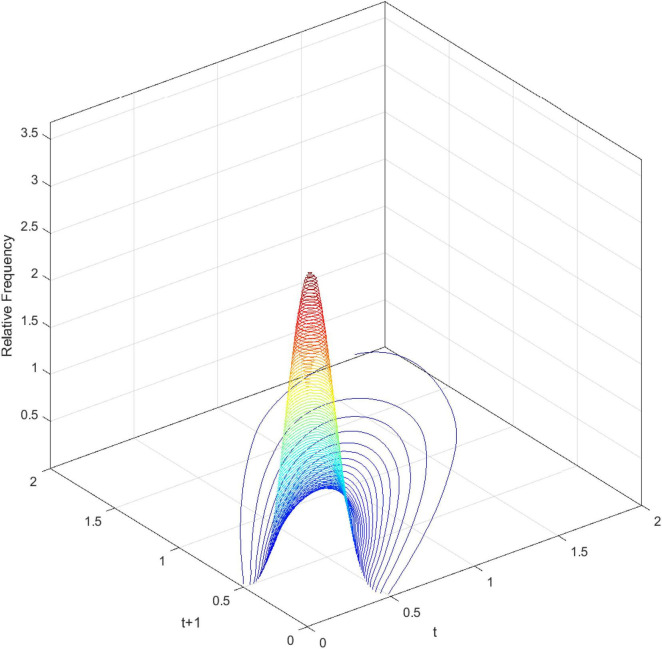
Three-dimensional plot of transition probability kernel for the relative RSVAPC of all countries with annual transitions. Source: Authors’ calculation.

**FIGURE 4:**
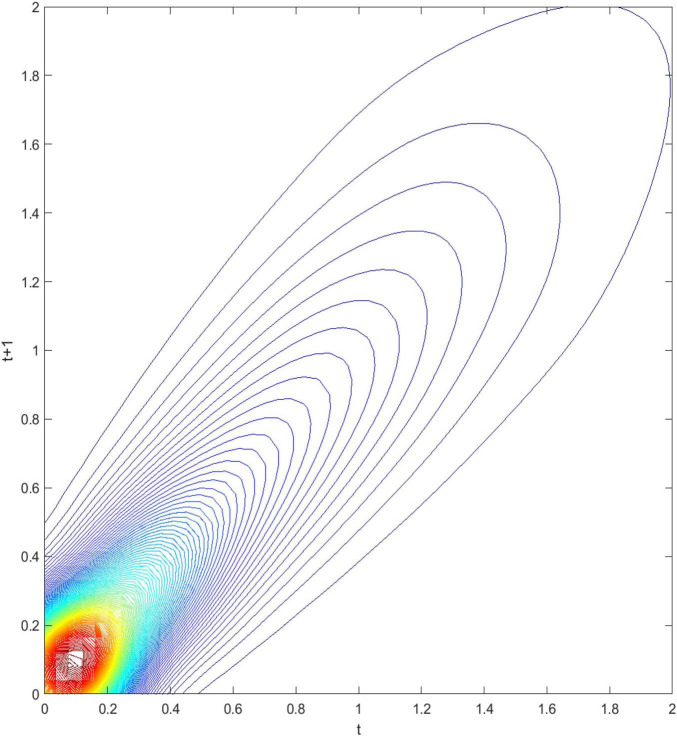
Contour map of transition probability kernel for the RSVAPC of all countries with annual transitions. Source: Authors’ calculation.

Figure 3 demonstrates the three-dimensional kernel-based transition probability for the Relative Service Value-Added per Capita (RSVAPC) of all countries. Secondly, Figure 4 presents a contour map along with the transition dynamics of the national units. In Figure 3, the relative frequency indicates the probability of transition at the country level from one particular RSVAPC value in year *t* to another RSVAPC value in year *t1*. It is noteworthy that the RSVAPC value is calculated based on the global average; hence, one is the average value. Consequently, a value less than one and larger than one marks a below-average and above-average RSVAPC, respectively. Figure 3 indicates that the majority of countries had an RSVAPC value of nearly 0.1, indicating a below-average RSVAPC.

In Figure 4, the contour map shows a top view of the three-dimensional graph. It can be visualized that Figure 4 could be separated into several vertical lines, with each line serving as a probability density function by itself—showing the probability of transitions from period *t*to period *t+1*. For instance, when a specific country has the RSVAPC value of 0.1 at period *t*, a vertical line intersecting the horizontal axis at 0.1 can be imagined. The intersection points of this imaginary vertical line with the contour lines illustrate the probability of climbing from the initial level of 0.1 to other values of RSVAPC plotted on the vertical axis. It can be seen from the graph that the imaginary vertical line peaks at the point where the RSVAPC value on the vertical axis is also 0.1. This indicates that a country with 0.1 as an initial value of RSVAPC will have a relatively high probability to remain at 0.1 in the following year. It can also be seen that the imaginary vertical line will reach the last contour line when the RSVAPC value on the vertical axis is equal to 0.65. This means that there is a low probability to climb from 0.1 to 0.65 and it is even lower when moving up to higher regions where contour lines are not visible.

In conclusion, the persistence is quite high, based on Figures 3, 4. Evidently, as shown in Figure 4, there is a very high probability for the countries to remain in their current states of service output capacity. The probability mass peaks along the diagonal, thereby showing that there is a very high probability for the countries to remain at their current positions. This illustrates very slow progress in the development of the service sector. Moreover, as the variability (the probability of moving upward/downward in the next period) in probability mass is relatively low for countries with RSVAPCs less than 0.4, perseverance is observed to be more severe for countries with lower values of RSVAPC. Thus, Figures 3, 4 clearly show that the development of the service sector in countries with extremely low values of RSVAPC remains sluggish, thereby indicating a disparity in the development patterns of the global service sector. This is the first piece of evidence for the unbalanced and inadequate development of the concurrent service sector. The disparity and rigidity inherent in the transition dynamics in both the figures will subsequently translate into a country-level long-run steady-state distribution of RSVAPC.

**FIGURE 5:**
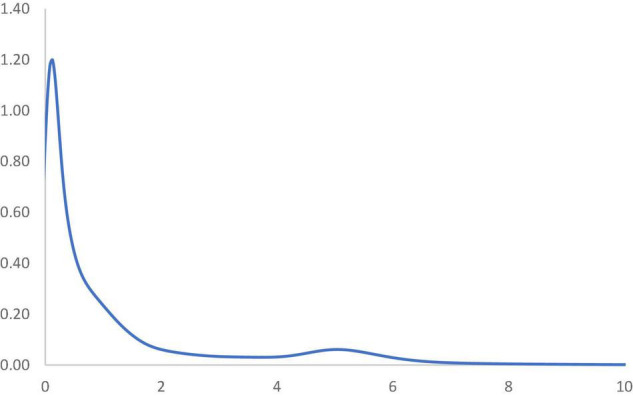
Ergodic distribution for the relative RSVAPC of all countries with annual transitions. Source: Authors’ calculation. N.B. The vertical axis indicates the density of probability and the horizontal axis indicates RSVAPC values.

Figure 5 shows the long-run steady-state ergodic distribution of the countries. As observed, several countries are likely to converge where the RSVAPC value is 0.08—the highest peak observed from the distribution. As one is the global average; industrial development in numerous countries will be very low if the transition dynamics do not change. It can also be observed that convergence club at a higher level, with a small peak where the value of RSVPAC value is 5.1. It indicates that these countries will congregate at this particular value of RSVAPC. Despite the higher values of RSVAPC as compared to the global average, the situation is unlikely to be impressive, since it suggests that many countries across the globe, with few exceptions, will incline toward low production capacity as a norm.

Considering that the service sector will fuel the future development of the service sector, the ideal overall situation to manage the issues of the low and uneven capacity of production would be a steady convergence to the global average. Eventually, countries with below-average values of RSVAPC will need to climb upward and/or the ones with above-average values to move downward. However, countries with high production capacity should not lower their output. As a consequence, there will a strong plea of attention to countries with below-average RSVAPC values, especially those with low or no capability to move up. This study helps in identifying these countries by observing the MPP.

**FIGURE 6:**
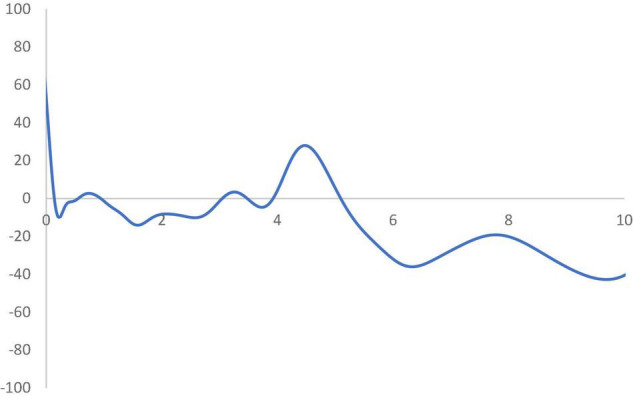
Mobility probability plot (MPP) for the RSVAPC of all countries. Source: Authors’ calculation. N.B. The vertical axis indicates net upward mobility (%) and the horizontal axis indicates RSVAPC values.

In Figure 6, the MPP plots the probability of net upward mobility, ranging from -100 to 100, as a percentage against the RSVAPC values. A positive value represents a country with a positive probability of net upward mobility, while a negative value illustrates a country with a negative probability of upward mobility. When moving from a region above the horizontal axis to the one below the horizontal axis, the MPP will intersect the horizontal axis. It is imperative to note that countries on the left-hand side of the intersection point have a positive value while the ones on the right-hand side of the intersection point have a negative value. Therefore, a large number of countries will converge around the point of intersection. Consequently, the transition dynamics underlying the MPP in Figure 6 enable a thorough understanding of the shape of the ergodic distribution in Figure 5. Figure 6 shows that the MPP intersects at the horizontal axis where the value of RSVAPC is 0.16. Subsequently, until the MPP intersects the horizontal axis again at the point where the value of RSVAPC value is 3.9, it remains negative. Therefore, within the distribution, only those countries with extremely low levels of output (RSVAPC values of less than 0.16) and extremely high levels of output (RSVAPC values of more than 3.9) showed a higher likelihood to move upward.

Based on this alarming result, one may think about the reason most, if not all, countries failing to make progress in their service sectors, while a few of them continue to outperform the rest. To examine the service sector growth in detail, the database is divided into the wealthier Global North and the poorer Global South.

### Comparison Between the Global North and the Global South

**FIGURE 7:**
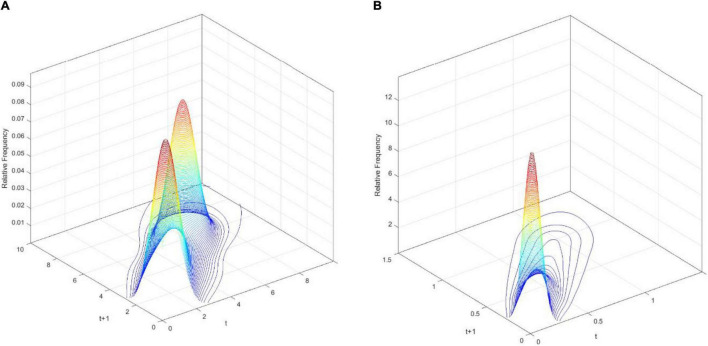
Three-dimensional plot of transition probability kernel for the relative RSVAPC of countries for the Global North **(A)** and the Global South **(B)** with annual transitions. Source: Authors’ calculation.

**FIGURE 8:**
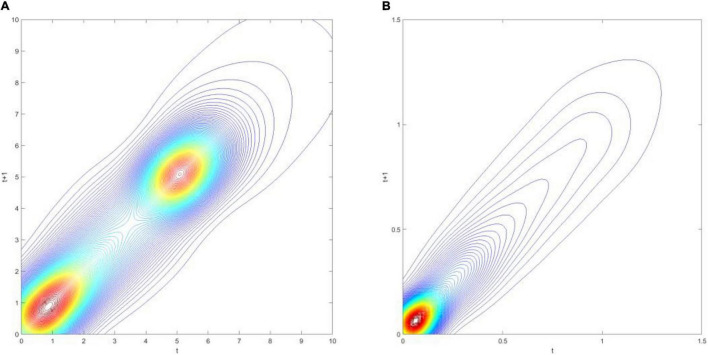
Contour map of transition probability kernel for the RSVAPC of countries for the Global North **(A)** and the Global South **(B)** with annual transitions. Source: Authors’ calculation.

In Figure 7, the three-dimensional kernel-based transition probabilities for RSVAPC of the two important regions (the Global North and the Global South) with annual transitions can be observed. Subsequently, Figure 8 shows the corresponding contour maps.

Figure 7A illustrates two peaks, situated at RSVAPC values of 1 and 5, in the three-dimensional graph for the Global North. This suggests that for most countries of the Global North, the development in the service sector was well above the global average with a distinct group of countries experiencing five times above the average production capacity. On the other hand, Figure 7B demonstrates a contrasting scenario. Here, the peak is located at an extremely low value of RSVAPC—0.05, which is also far below the global average. This shows severe underdevelopment in most countries of the Global South, thereby indicating a disparity in the degree of global service development.

A larger variability (i.e., the likelihood of moving upward/downward in the following period) in the Global North as compared to the Global South can be observed in Figures 8A,B. Moreover, a rise in production capacity in both the Global North and the Global South leads to an increase in variability. It demonstrates that rigidity is relatively pronounced in countries with low values of RSVAPC—a key source of unbalanced development.

**FIGURE 9:**
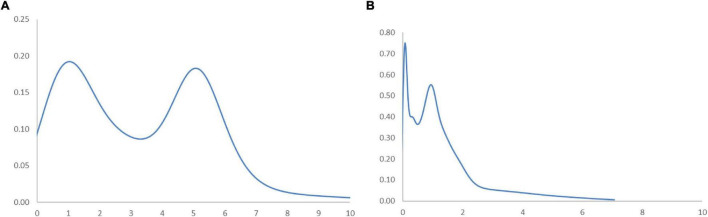
Ergodic distributions for the RSVAPC of countries for the Global North **(A)** and the Global South **(B)** with annual transitions. Source: Authors’ calculation. N.B. The vertical axis indicates the density of probability and the horizontal axis indicates RSVAPC values.

Figure 9 shows the transition dynamics translating into the long-run steady-state ergodic distribution. From Figure 9A, it can be observed that the two most obvious peaks are situated at the RSVAPC values of 1.35 and 5.18. This suggests an encouraging future development in the service sector of the Global North. Meanwhile, Figure 9B illustrates that the two peaks appear at RSVAPC values of 0.09 and 0.9, respectively. This finding is considered disturbing due to its demonstration that most countries of the Global South will have extremely low levels of RSVAPC relative to future development in the service sector. Moreover, the ergodic distribution is more scattered in the Global North as compared to the Global South. It indicates that countries are mostly tied and focused on the two below-the-average peaks with a few exceptions in the latter.

**FIGURE 10:**
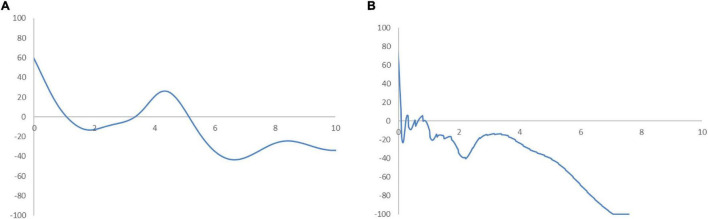
Mobility probability plot (MPP) for the RSVAPC of countries for the Global North **(A)** and the Global South **(B)**. Source: Authors’ calculation. N.B. The vertical axis indicates net upward mobility (%) and the horizontal axis indicates RSVAPC values.

In Figure 10, the MPP suggests a reason behind the current forms of the ergodic distributions. It can be observed from Figure 10A that the Global North countries with an RSVAPC value of less than 1.13 have a positive net probability of upward mobility. At this intersection, the first peak in the ergodic distribution remains evident. The second intersection point presents an RSVAPC situated at level 3.5, representing a second peak in the ergodic distribution. It is worth noting that these two peaks appear above the global average. Consequently, the MPP shows the influence of transition dynamics on a higher development in the service sector of the Global North. Conversely, in Figure 10B, the entire MPP appeared almost below the horizontal axis which suggests a negative net probability of upward mobility. The first intersection point is situated where the RSVAPC value is 0.09, which is far below the former first intersection point (i.e., RSVAPC value of 1.13). It reflects that the Global South countries have relatively low service output than the global average. It is also important to note that the MPP is positive for the RSVAPC values between 0.2 and 0.9, leading to the emergence of another peak in the ergodic distribution. This is due to the presence of a few countries such as China in the Global South with a high production capacity and upward mobility. However, these exceptions are unlikely to provide any help for the development of the overall service sector in the Global South. Without such sporadic cases of countries with an RSVAPC value of less than 0.09, the entire MPP of the Global South remains below the horizontal axis. It implies that only the Global South countries with extremely low service output (those countries with an RSVAPC value less than 0.09) have a chance for upward mobility. Additionally, countries that have RSVAPCs greater than 0.09 will fail to maintain progress in their service development.

The alarming findings suggest a possibility of increasing disparity between the Global North and the Global South in the future. The development of the service sector in the Global North will continue to make greater strides, while the output capacity in many Global South countries struggles to reach the global average. This prompts the need for a re-examination of the development policy in most countries of the Global South. Moreover, the governments are required to develop pragmatic policies to address and remove a large number of obstacles (e.g., to reduce transaction costs) to the service sector development to bridge the gap between the two regions.

### Comparison Between Countries Based on Income Groups (World Bank Classification)

**FIGURE 11:**
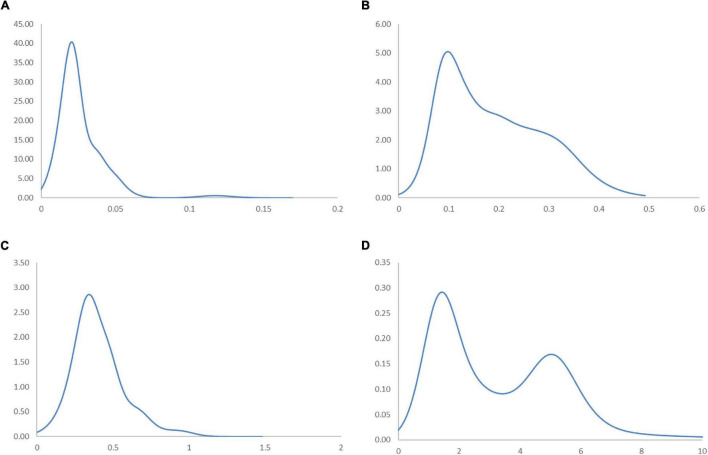
Ergodic distributions for the RSVAPC of countries for different income groups of countries with annual transitions. Source: Authors’ calculation. N.B. The vertical axis indicates the density of probability and the horizontal axis indicates RSVAPC values. **(A)** Low-income countries; **(B)** Lower-middle income countries; **(C)** Upper-middle income countries; **(D)** High-income countries.

**FIGURE 12:**
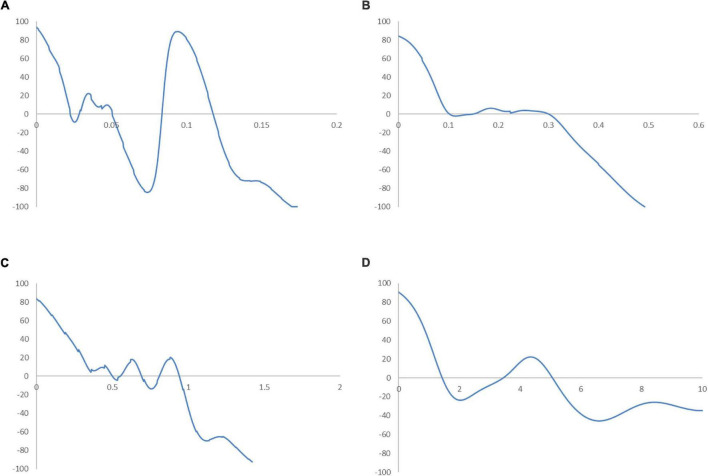
Mobility probability plot (MPP) for the RSVAPC of countries for different income groups of countries. Source: Authors’ calculation. N.B. The vertical axis indicates net upward mobility (%) and the horizontal axis indicates RSVAPC values. **(A)** Low-income countries; **(B)** Lower-middle income countries; **(C)** Upper-middle income countries; **(D)** High-income countries.

To assess the correlation between income and industrial development without the influence of sporadic cases in the Global South, the ergodic distributions for different income groups of countries are considered. The ergodic distributions and the MPP for four respective income groups are shown in Figures 11, 12.

In Figure 11A, the entire ergodic distribution of low-income countries is compressed to the left of the global average, with convergence clubs at the levels of 0.02, 0.04, and 0.12. Owing to the highly concentrated distribution, the peaks are visibly pronounced, and the convergence clubs are closely situated. In Figure 12A, the MPP for the low-income countries indicates that countries have the chance to move upward, only when the values of RSVAPC are less than 0.02, between 0.03 and 0.05, and between 0.08 and 0.12. When the RSVAPC value reaches 0.17, the net probability of upward mobility is at -100. Accordingly, low-income countries are unable to transit through the threshold where the RSVAPC value is 0.17. Upon reaching this threshold, a level less than half of the global average, the RSVAPC value of the country will decline, thereby moving it downward in the distribution. This suggests the possibility of a service development trap in low-income countries. It is an alarming situation, as development in the service sector is the future growth engine in both developed and developing countries. For low-income countries, development in the service sector is the major pathway to achieve a reasonable living standard in the future. These study findings imply that a country continues to face poverty if it exists unless there is external interference.

Figure 11B shows the ergodic distribution for the lower-middle-income countries. The distribution is more dispersed than that of low-income countries, where all the convergence clubs—0.1, 0.22, and 0.31—are below the global average. This shows that the development of the service sector in lower-middle-income countries is far from encouraging. In Figure 12B, the MPP confirms the implications of the ergodic distribution for the lower-middle-income countries. The first intersection point appears where the level of RSVAPC is very low at 0.1. The second and the third range of positive net upward mobility appear where the levels of RSVAPC are approximately 0.15–0.22 and 0.23–0.31, respectively. When the value of RSVAPC reaches 0.5, the net probability of upward mobility becomes -100. In other words, the lower-middle-income countries with no or relatively low levels of service output have a better chance of making proper progress in service development. However, if the output level is close to one-third of the global average (i.e., an RSVAPC level of 0.31), they struggle to maintain progress in their development. Moreover, when the level of RSVAPC for lower-middle-income countries is 0.5, the net upward mobility reaches -100. It suggests that a decline in an RSVAPC level of a country to 0.5, will bring the mobility downward in the distribution. Although a negative net upward mobility appears at a higher output level in low-income countries, a development trap in lower-middle-income countries can be found. After attaining a slightly higher level of economic growth, these countries fail to continue making further progress in development required for sustainable growth.

In upper-middle-income countries, the situation appears to be better. Figure 11C shows three convergence clubs. The first two peaks are below the global average at 0.37 and 0.7, respectively. However, the third peak appears at level 0.98, which is close to the global average. Despite its similarity with the low-income and lower-middle-income countries, the overall shape of the ergodic distribution for the upper-middle countries is not comparable with the previous two distributions, as it is more dispersed and has a higher average level of RSVAPC. In Figure 12C, the MPP for the upper-middle-income countries shows a positive net upward mobility for RSVAPC values ranging from 0.8 to 0.95. It suggests some upper-middle-income countries with RSVAPC values around the global average can maintain sustainable development. However, the net upward mobility becomes -100 when the value of RSVAPC reaches 1.45. To avoid the development trap, the upper-middle-income countries are required to reach and sustain a high rate of development.

In Figure 11D, the first peak of the ergodic distribution for high-income countries is situated at the RSVAPC level of 1.7, while the second peak is situated at 5.2. Looking at this distribution, many high-income countries will have industrial development above the global average. Moreover, some of the countries even have five times the production capacity of the rest of the world. The MPP in Figure 12D shows that the first intersection point appears when the level RSVAPC level is at 1.5, while the second intersection point appears when the level of RSVAPC level is at 3.5. These two intersection points provide the two convergence clubs situated in the ergodic distribution. Unlike the previous MPPs for the three different income groups, there is no obvious development trap within the high-income countries. The most negative net upward mobility is about -50 when the level of RSVAPC reaches 6.7.

In the conclusion of the comparison based on four different income groups, countries with higher per capita income will perform better in the development of their service sector than those with low per capita income. Based on the distribution dynamic analysis, the growth of the service sector is linked with per capita income, thereby creating demand for services within the market. More importantly, based on the ergodic distribution and the MPP analysis, various underdeveloped countries lack the ability for upward mobility in the distribution without any external interferences. Therefore, it is not a natural and inevitable process for a specific country to evolve from agricultural to industrial and then to a service economy.

### Comparison Between Regions

**FIGURE 13:**
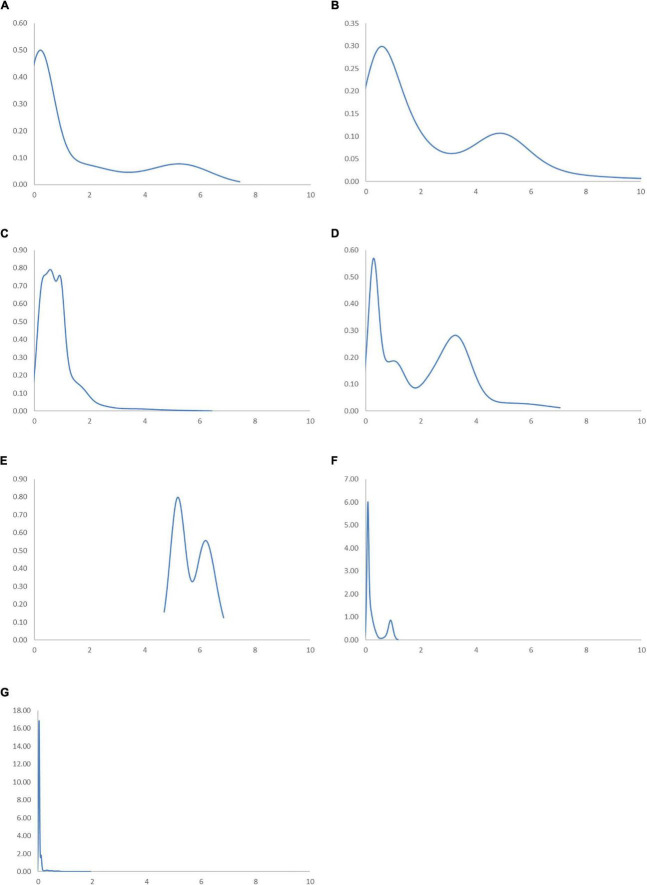
Ergodic distributions for the RSVAPC of countries for different geographical regions with annual transitions. Source: Authors’ calculation. N.B. The vertical axis indicates the density of probability and the horizontal axis indicates RSVAPC values. **(A)** East Asia and Pacific; **(B)** Europe and Central Asia; **(C)** Latin America and the Caribbean; **(D)** The Middle East and North Africa; **(E)** North America; **(F)** South Asia; **(G)** Sub-Saharan Africa.

**FIGURE 14:**
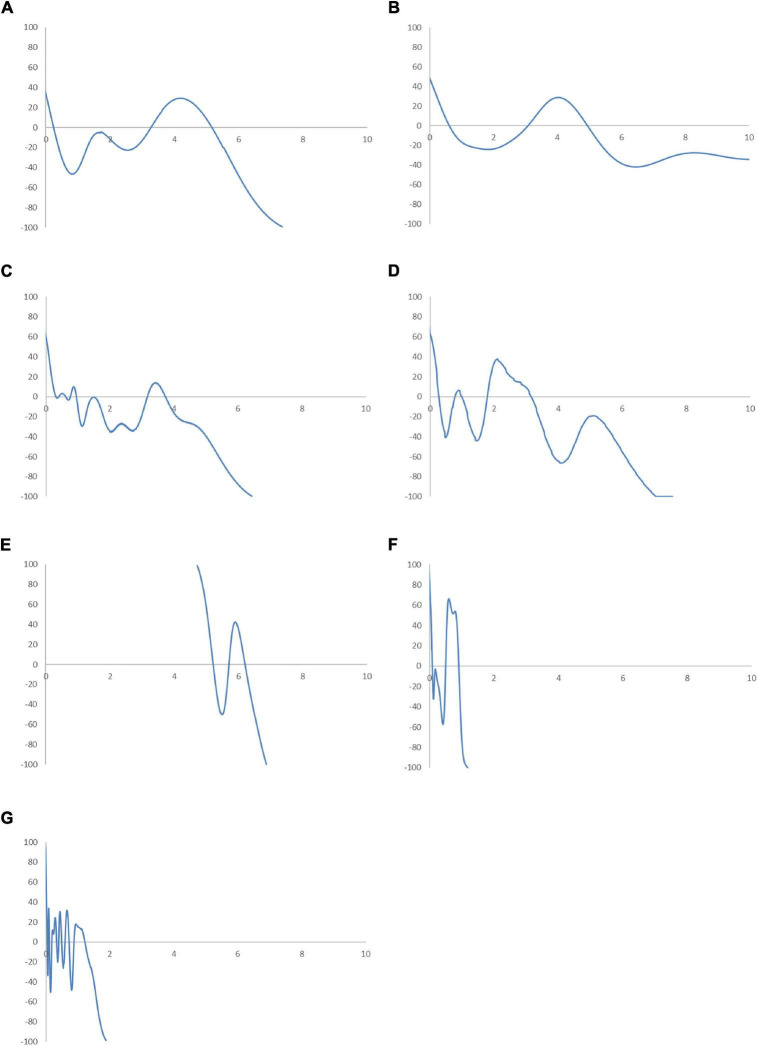
Mobility probability plot (MPP) for the RSVAPC of countries for different geographical regions. Source: Authors’ calculation. N.B. The vertical axis indicates net upward mobility (%) and the horizontal axis indicates RSVAPC values. **(A)** East Asia and Pacific; **(B)** Europe and Central Asia; **(C)** Latin America and the Caribbean; **(D)** The Middle East and North Africa; **(E)** North America; **(F)** South Asia; **(G)** Sub-Saharan Africa.

Figures 13, 14 illustrate regional ergodic distributions and MPP, respectively. There are similarities between the ergodic distributions for the East Asia and Pacific region, the Europe and Central Asia region, the Latin America and Caribbean region, and the Middle East and North Africa region. From the ergodic distributions for these four regions, it can be observed that numerous countries cluster around the levels of RSVAPC which is less than half of the global average, while a few countries cluster around the levels of RSVAPC above the global average. For these regions, the MPP confirms the implications of their ergodic distribution. The MPP of these regions shows that the first intersection points are situated below one-half of the global average. Additionally, they have some positive net mobility when they reach a higher level of RSVAPC.

The progress of development remains the lowest in two particular regions—the Sub-Saharan Africa region and the South Asia region. For these regions, the ergodic distributions are far below the RSVAPC value of 2. Moreover, the ergodic distribution for the Sub-Saharan Africa region is the most compact, indicating the two pronounced peaks at levels 0.02 and 0.12, respectively. In the South Asia region, the two peaks are slightly higher—0.08 and 0.95—as compared to that of the Sub-Saharan Africa region. However, when the value of RSVAPC value reaches 1.87 in the Sub-Saharan Africa region and 1.17 in the South Asia region, the net upward mobility value becomes -100. It implies that despite slightly better development progress in the South Asia region as compared to the Sub-Saharan Africa region, the former will encounter a development trap with a lower value of RSVAPC.

Compared to all the regions, North America has a relatively unique ergodic distribution and MPP. For this region, the ergodic distribution appears above an RSVAPC value of 4.7 and the first intersection point for the MPP is situated at 5.2. Moreover, it is the only region where the RSVAPC of all countries is above the global average. It is worth noting that the region will face from development trap. The net upward mobility reaches -100 in the North America region when the value of RSVAPC reaches 6.8. While the development traps in the Sub-Saharan Africa region create a hindrance in service development, the development trap in the North American region will delay development breakthroughs of the service sector across the world.

Based on the regional evidence, the primary peaks of the ergodic distributions for most regions are far below the global average, except North America. This indicates that almost all regions will not attain convergence to the global mean. The convergence clubs in all regions suggest that the unequal industrial development at the regional level is universal. The focus must be placed on the removal of upward-moving obstacles for countries that have a low level of RSVAPC and negative net upward mobility.

## Conclusion and Implications

This paper aims to examine the transitional dynamics of the globe’s service output by using a new framework of distribution dynamics analysis, namely, the mobility probability plot (MPP). It is the first study to investigate the convergence and dynamics of service value-added per capita in the world. With the consideration of heterogeneity in service value-added per capita, the results are useful to policy makers in identifying the key groups for priority interventions.

It was found that, in the all-countries analysis, only those countries with extremely low levels of output (RSVAPC values of less than 0.16) and extremely high levels of output (RSVAPC values of more than 3.9) showed a higher likelihood to move upward. For the comparison between the Global North and the Global South, it was found that the development of the service sector in the Global North will continue to make greater strides, while the output capacity in many Global South countries struggles to reach the global average. However, for the comparison based on four different income groups, it was found that countries with higher per capita income will perform better in the development of their service sector than those with low per capita income. Finally, this study shows that the Sub-Saharan Africa region and the South Asia region are both very important in the alleviation of global inequality.

The COVID-19 could exacerbated the inequality in service sector in the future compare with the general situation. Based on this situation, the following policy implications can be drawn based on the empirical findings: first, policy makers need to pay more attention to the countries that have an RSVAPC level below the global average. Service development planning and policy measures need to be implemented for the countries have low RSVAPC level. Second, the global north countries should help the global south countries to achieve more balanced and inclusive growth for the service sector so as to meet the United Nations’ sustainable development goals. Finally, more support should be provided to those underdeveloped countries (especially for those located in Sub-Saharan Africa and the South Asia regions) in a more targeted manner through technical cooperation with developed countries.

## Data Availability Statement

Publicly available datasets were analyzed in this study. This data can be found here: https://databank.worldbank.org/reports.aspx?source=global-financial-development&Type=METADATA&preview=on#.

## Author Contributions

NM, WS, and TC: conceptualization, data curation, methodology, software, visualization, writing — original draft preparation, and writing — review and editing. TH: conceptualization, project administration, data curation, writing — original draft preparation, and writing — review and editing. All authors contributed to the article and approved the submitted version.

## Conflict of Interest

The authors declare that the research was conducted in the absence of any commercial or financial relationships that could be construed as a potential conflict of interest.

## Publisher’s Note

All claims expressed in this article are solely those of the authors and do not necessarily represent those of their affiliated organizations, or those of the publisher, the editors and the reviewers. Any product that may be evaluated in this article, or claim that may be made by its manufacturer, is not guaranteed or endorsed by the publisher.

## References

[B1] Alcántara V. Padilla E. (2009). Input–output subsystems and pollution: an application to the service sector and CO2 emissions in Spain. *Ecol. Econ.* 68 905–914.

[B2] Álvarez-Rodríguez C. Martín-Gamboa M. Iribarren D. (2019). Combined use of data envelopment analysis and life cycle assessment for operational and environmental benchmarking in the service sector: a case study of grocery stores. *Sci. Total Environ.* 667 799–808. 10.1016/j.scitotenv.2019.02.433 30851613

[B3] Antonelli C. Tubiana M. (2020). Income inequality in the knowledge economy. *Struct. Change Econ. Dyn.* 55 153–164.

[B4] Basuil D. A. Datta D. K. (2019). Effects of firm-specific and country-specific advantages on relative acquisition size in service sector cross-border acquisitions: an empirical examination. *J. Int. Manag.* 25 66–80. 10.1016/j.intman.2018.07.001

[B5] Beqiraj E. Fanti L. Zamparelli L. (2019). Sectoral composition of output and the wage share: the role of the service sector. *Struct. Change Econ. Dyn.* 51 1–10. 10.1016/j.strueco.2019.06.009

[B6] Bosworth B. Collins S. M. (2008). Accounting for growth: comparing China and India. *J. Econ. Perspect.* 22 45–66. 10.1257/jep.22.1.45

[B7] Brown P. Ly T. Pham H. Sivabalan P. (2020). Automation and management control in dynamic environments: managing organisational flexibility and energy efficiency in service sectors. *Br. Account. Rev.* 52:100840.

[B8] Butnar I. Llop M. (2011). Structural decomposition analysis and input–output subsystems: changes in CO2 emissions of Spanish service sectors (2000–2005). *Ecol. Econ.* 70 2012–2019. 10.1016/j.ecolecon.2011.05.017

[B9] Cavelaars P. (2006). Output and price effects of enhancing services sector competition in a large open economy. *Eur. Econ. Rev.* 50 1131–1149.

[B10] Chakraborty C. Nunnenkamp P. (2008). Economic reforms, FDI, and economic growth in India: a sector level analysis. *World Dev.* 36 1192–1212. 10.1016/j.worlddev.2007.06.014

[B11] Cheong T. S. Li V. J. Shi X. (2019). Regional disparity and convergence of electricity consumption in China: a distribution dynamics approach. *China Econ. Rev.* 58 101154. 10.1016/j.chieco.2018.02.003

[B100] Cheong T. S. Wu Y. (2018). Convergence and transitional dynamics of China’s industrial output: a county-level study using a new framework of distribution dynamics analysis. *China Econ. Rev.* 48, 125–138. 10.1016/j.chieco.2015.11.012

[B12] Cheong T. S. Wu Y. Wu J. (2016). Evolution of carbon dioxide emissions in chinese cities: trends and transitional dynamics. *J. Asia Pacific Econ.* 21 357–377.

[B13] de Souza K. B. de Andrade Bastos S. Q. Perobelli F. S. (2016). Multiple trends of tertiarization: a comparative input–output analysis of the service sector expansion between Brazil and United States. *EconomiA* 17 141–158.

[B14] Doytch N. Uctum M. (2019). Spillovers from foreign direct investment in services: evidence at sub-sectoral level for the Asia-Pacific. *J. Asian Econ.* 60 33–44. 10.1016/j.asieco.2018.10.003

[B15] Fang C. Y. Hu J. L. Lou T. K. (2013). Environment-adjusted total-factor energy efficiency of Taiwan’s service sectors. *Energy Policy* 63 1160–1168. 10.1016/j.enpol.2013.07.124 32287870 PMC7115794

[B16] Freytag A. Fricke S. (2017). Sectoral linkages of financial services as channels of economic development—an input–output analysis of the Nigerian and Kenyan economies. *Rev. Dev. Finance* 7 36–44.

[B17] García-Pozo A. Marchante-Mera A. J. Campos-Soria J. A. (2018). Innovation, environment, and productivity in the Spanish service sector: an implementation of a CDM structural model. *J. Cleaner Prod.* 171 1049–1057. 10.1016/j.jclepro.2017.10.087

[B18] Goodell J. W. (2020). COVID-19 and finance: agendas for future research. *Finance Res. Lett.* 35:101512. 10.1016/j.frl.2020.101512 PMC7152896 32562472

[B19] Gowdy J. M. Miller J. L. (1987). Energy use in the US service sector: an input-output analysis. *Energy* 12 555–562. 10.1016/0360-5442(87)90096-x

[B20] Haller S. A. Lyons S. (2019). Effects of broadband availability on total factor productivity in service sector firms: evidence from Ireland. *Telecommun. Policy* 43 11–22. 10.1016/j.telpol.2018.09.005

[B21] Hassan K. Abdullah A. (2015). Effect of oil revenue and the Sudan economy: econometric model for services sector GDP. *Proc. Soc. Behav. Sci.* 172 223–229. 10.1016/j.sbspro.2015.01.358

[B22] Hou H. Wang J. Yuan M. Liang S. Liu T. Wang H. (2020). Estimating the mitigation potential of the Chinese service sector using embodied carbon emissions accounting. *Environ. Impact Assess. Rev.* 86 106510. 10.1016/j.eiar.2020.106510

[B23] Karim N. H. Rahman N. S. F. A. Shah S. F. S. S. J. (2018). Empirical evidence on failure factors of warehouse productivity in Malaysian logistic service sector. *Asian J. Shipping Logist.* 34 151–160. 10.1016/j.ajsl.2018.06.012

[B24] Kinfemichael B. Morshed A. M. (2018). Unconditional convergence of labor productivity in the service sector. *J. Macroecon.* 59 217–229.

[B101] Kühn S. Milasi S. Yoon S. (2018). *World Employment and Social Outlook: Trends 2018*. Geneva: International Labour Organization (ILO).

[B25] Kuijs L. Wang T. (2006). China’s pattern of growth: moving to sustainability and reducing inequality. *China World Econ.* 14 1–14.

[B26] Latorre M. C. Yonezawa H. Zhou J. (2018). A general equilibrium analysis of FDI growth in Chinese services sectors. *China Econ. Rev.* 47 172–188.

[B27] Lee J. W. McKibbin W. J. (2018). Service sector productivity and economic growth in Asia. *Econ. Modelling* 74 247–263.

[B28] Liao J. (2020). The rise of the service sector in China. *China Econ.Rev.* 59 101385.

[B29] Lin B. Zhang G. (2017). Energy efficiency of Chinese service sector and its regional differences. *J. Cleaner Prod.* 168 614–625.

[B30] Mehta A. Hasan R. (2012). The effects of trade and services liberalization on wage inequality in India. *Int. Rev. Econ. Finance* 23 75–90.

[B31] Miroudot S. Sauvage J. Shepherd B. (2012). Trade costs and productivity in services sectors. *Econ. Lett.* 114 36–38. 10.1016/j.econlet.2011.09.005

[B32] Mookerjee R. Kalipioni P. (2010). Availability of financial services and income inequality: the evidence from many countries. *Emerg. Mark. Rev.* 11 404–408. 10.1016/j.ememar.2010.07.001

[B33] Morikawa M. (2019). Innovation in the service sector and the role of patents and trade secrets: evidence from Japanese firms. *J. Jpn. Int. Econ.* 51 43–51. 10.1016/j.jjie.2018.10.003

[B34] Mulder P. De Groot H. L. Pfeiffer B. (2014). Dynamics and determinants of energy intensity in the service sector: a cross-country analysis, 1980–2005. *Ecol. Econ.* 100 1–15.

[B35] Nabar M. M. Yan M. K. (2013). *Sector-Level Productivity, Structural Change, And Rebalancing in China.* Washington, DC: International Monetary Fund.

[B36] National Health Commission [NHC] (2021). National Health Commission (NHC) of the People’s Republic of China. Available online at: http://www.nhc.gov.cn/ (accessed October 08, 2021).

[B37] Noland M. Park D. Estrada G. B. (2012). *Developing The Service Sector As Engine Of Growth For Asia: An Overview.* Asian Development Bank Economics Working Paper Series, 320. Manila: Asian Development Bank.

[B38] Ogundele O. J. Pavlova M. Groot W. (2020). Socioeconomic inequalities in reproductive health care services across Sub-Saharan Africa. a systematic review and meta-analysis. *Sex. Reprod. Healthc.* 25 100536. 10.1016/j.srhc.2020.100536 32526462

[B39] Omri A. (2018). Entrepreneurship, sectoral outputs and environmental improvement: international evidence. *Technol. Forecast. Soc. Change* 128 46–55. 10.1016/j.techfore.2017.10.016

[B40] Quah D. (1993a). Empirical cross-section dynamics in economic growth. *Eur. Econ. Rev.* 37 426–434. 10.1016/0014-2921(93)90031-5

[B41] Quah D. (1993b). Galton’s fallacy and tests of the convergence hypothesis. *Scand. J. Econ.* 95 427–443.

[B42] Quah D. (1996a). Empirics for economic growth and convergence. *Eur. Econ. Rev.* 40 1353–1375.

[B43] Quah D. (1996b). Regional convergence clusters across Europe. *Eur. Econ. Rev.* 40 951–958. 10.1016/0014-2921(95)00105-0

[B44] Quah D. (1996c). Twin peaks: growth and convergence in models of distribution dynamics. *Econ. J.* 106 1045–1055.

[B45] Quah D. (1997). Empirics for growth and distribution: stratification, polarization, and convergence clubs. *J. Econ. Growth* 2 27–59.

[B46] Sauian M. S. Kamarudin N. Rani R. M. (2013). Labor productivity of services sector in malaysia: analysis using input-output approach. *Proc. Econ. Finance* 7 35–41.

[B47] Shi X. Yu J. Cheong T. S. (2020). Convergence and distribution dynamics of energy consumption among China’s households. *Energy Policy* 142 111496. 10.1016/j.enpol.2020.111496

[B102] Silverman B. W. (1986). *Density Estimation for Statistics and Data Analysis*. New York, NY: Chapman and Hall.

[B48] Singh R. S. (2014). India’s service sector–shaping future of indian retail industry. *Proc. Econ. Finance* 11 314–322. 10.1016/s2212-5671(14)00199-3

[B49] Tang C. F. Shahbaz M. (2013). Sectoral analysis of the causal relationship between electricity consumption and real output in Pakistan. *Energy Policy* 60 885–891. 10.1016/j.enpol.2013.05.077

[B50] Tochkov K. Yu W. (2013). Sectoral productivity and regional disparities in China, 1978–2006. *Comp. Econ. Stud.* 55 582–605. 10.1057/ces.2013.19

[B51] Voulis N. Warnier M. Brazier F. M. (2017). Impact of service sector loads on renewable resource integration. *Appl. Energy* 205 1311–1326. 10.1016/j.apenergy.2017.07.134

[B52] Wang J. Zhang K. (2014). Convergence of carbon dioxide emissions in different sectors in China. *Energy* 65 605–611. 10.1016/j.energy.2013.11.015

[B53] Wang R. Hao J. X. Wang C. Tang X. Yuan X. (2020). Embodied CO2 emissions and efficiency of the service sector: evidence from China. *J. Cleaner Prod.* 247:119116. 10.1016/j.jclepro.2019.119116

[B54] World Bank (2021). *World Bank National Accounts Data, And OECD National Accounts Data Files.* Washington, DC: Word Bank.

[B55] Wu H. X. (2007). Measuring productivity performance by industry in China, 1980-2005. *Int. Prod. Monit.* 15 55–74.

[B56] Xiao H. Shan Y. Zhang N. Zhou Y. Wang D. Duan Z. (2019). Comparisons of CO2 emission performance between secondary and service industries in Yangtze River Delta cities. *J. Environ. Manag.* 252:109667. 10.1016/j.jenvman.2019.109667 31627097

[B57] Xing R. Hanaoka T. Kanamori Y. Masui T. (2018). Estimating energy service demand and CO2 emissions in the Chinese service sector at provincial level up to 2030. *Resour. Conserv. Recycling* 134 347–360. 10.1016/j.resconrec.2018.02.030

[B58] Zhang G. Lin B. (2018). Impact of structure on unified efficiency for Chinese service sector—a two-stage analysis. *Appl. Energy* 231 876–886. 10.1016/j.apenergy.2018.09.033

[B59] Zhang H. Shi X. Cheong T. S. Wang K. (2020). Convergence of carbon emissions at the household level in China: a distribution dynamics approach. *Energy Econ.* 92:104956. 10.1016/j.eneco.2020.104956

